# Thermal Characteristics of Breast Surface Temperature in Healthy Women

**DOI:** 10.3390/ijerph18031097

**Published:** 2021-01-26

**Authors:** Anna Lubkowska, Monika Chudecka

**Affiliations:** 1 Chair and Department of Functional Diagnostics and Physical Medicine, Faculty of Health Sciences, Pomeranian Medical University, 54 Żołnierska, 71-210 Szczecin, Poland; anna.lubkowska@pum.edu.pl; 2 Institute of Physical Culture Sciences, Faculty of Physical Education and Health, University of Szczecin, 71-065 Szczecin, Poland

**Keywords:** infrared thermography, breast, surface temperatures

## Abstract

Thermography is widely used in the medical field, including in the detection of breast disorders. The aim of the research was to characterize the range of breast surface temperature values, taking into account the entire area of the mammary gland and, independently, the nipple, in healthy women. An additional aim was to assess the symmetry of the breast temperature distribution (using an IR camera) and the correlation of temperatures with the content of adipose tissue. Thermograms were made for the right and left breasts, each time delineating the area of the entire breast and a separate area of the nipple, chest, and abdomen. Analyzing the intergroup differences in temperature of selected body areas (T_mean_), it was shown that, in all cases, they were significantly higher in younger women. Statistical analysis showed no significant differences between breast and nipple temperatures in relation to the body sides. The highest temperatures within the mammary gland were recorded for the nipple area. The use of the high-resolution digital infrared thermal imaging method in early and screening preventive diagnoses of changes in the mammary gland requires individual interpretation of the results, taking into account the assessment of the physiological pattern of temperature distribution in both breasts.

## 1. Introduction

Infrared thermography is a method that allows researchers to assess temperature distribution on the body surface in terms of visualization of the heat radiated from the body.

Body surface temperature can be described as a complex map of isotherms, with a very wide range of temperatures changing under the influence of endo- and exogenous factors. Increased temperature of tissues is frequently the first symptom of pathological changes, even before the occurrence of structural or functional changes [1]. One of the main factors affecting the skin surface temperature under thermal comfort conditions is vascular control. The subcutaneous region contains the venous plexus, strongly affecting skin temperature and heat transfer from the skin to the environment. Recently, it has been shown that a high-resolution thermal image can provide important information about the complex thermoregulation system of the body [2].

The female breast, located on the anterior surface of the chest wall, consists mainly of adipose tissue containing glands responsible for the production of milk. Each breast has approximately 15 to 25 lobes arranged radially around the nipple. The most important element of the breast is the mammary gland, to which the milk ducts lead. The complex structure of the breast, undergoing changes in the course of ontogenesis, the composition and density of the tissue, as well as the size and various blood supply, all affect the diagnostic difficulties of changes within this gland, despite many currently available methods (ultrasonography (USG), ultrasonography with sonoelastography, mammography (MMG), magnetic resonance imaging (NMR), contrast agent and positron emission tomography (PET)) [3].

In the last 10 years, infrared thermography has proven to be a promising technique for early diagnosis of breast pathologies. Currently, attention is focused on the diagnostic importance of combining thermography with other types of examination in order to increase sensitivity and specificity and eliminate weaknesses of individual diagnostic methods. Several papers on the use of infrared thermal imaging for breast screening can be found in the current medical literature [4,5,6,7].

Thermography was approved as an adjunctive tool to other screening techniques in 1982 for use alongside a primary test, like mammography, by the U.S. Food and Drug Administration (FDA).

It is a functional examination aimed at looking for changes in the temperature distribution on the breast surface caused by, among other things, metabolic activity, and to first establish the surface isotherm pattern of the healthy breast. In healthy subjects, the pattern of skin surface temperature in the same body area on contralateral sides of the body is regarded by many as being very similar. The terms thermal symmetry and thermal asymmetry have arisen from this, the latter often being used to help medical practitioners make a diagnosis [8].

For an appropriate interpretation of an individual thermographic profile, it is necessary to establish a reference thermal pattern for each population group (for example, by age, race, or a specific area of the body). Having a thermal reference allows us to evaluate normal skin temperature (T_skin_) in different body segments and the proportion of bilateral body areas (side to side). Based on existing thermal maps and recognized standards for the interpretation of thermal imaging tests, it was assumed that a ΔT_skin_ greater than 0.5 °C between two symmetrical body sides strongly indicates a functional disorder in the assessed regions of interest, which may be the result of pathological conditions in tissues [9,10,11]. Our previous research on the diagnostic application of thermography focused on the determination of thermal maps of various areas of the body, taking into account age, sex, body structure, composition, and nutritional status [10,12,13,14]. 

With the increased use of thermal imaging, there is a need to have regulations and standards to provide accurate and consistent results as far as the diagnosis of changes in the breast area is concerned. Practical research work in this area was undertaken by The Department of Biotechnology of Tripura University and Jadavpur University (DBT-TU-JU), creating a database of breast thermograms, with 1100 breast thermograms of 100 subjects and taking into account the results of other imaging methods [15]. There has been an ongoing discussion in the literature on the usefulness of thermal imaging in the early diagnosis of breast cancer, the basis of which are results proving high sensitivity for thermography in the detection of breast cancers, and, at the same time, the difficulties in standardization of tests and interpretation of isotherm analysis results [4,16].

The aim of this the study was to characterize the distribution and range of breast surface temperature values, taking into account the entire area of the mammary gland, and, independently, for the nipple, in healthy women, which can constitute reference data for the process of medical diagnosis and prevention. An additional aim of the research was to assess the symmetry of the breast temperature distribution and the correlation of these temperatures with the content of adipose tissue. The research was carried out as a comparative study in two age groups of women, differing in the degree of mammary gland development.

## 2. Material and Methods

The research was conducted at the Department of Functional Diagnostics and Physical Medicine of the Pomeranian Medical University in Szczecin in the period from May to September 2020. The study included 105 healthy women aged 20–40 years. Each participant gave written consent to take part in the study according to the Declaration of Helsinki. This study was approved by the local ethics committee of the District Medical Chamber in Szczecin (Ref. No. 10/KB/VI/2018). The inclusion criteria for the study were: informed consent to participate, regular menstruation, the current absence of pregnancy, and the absence of changes in the mammary gland confirmed by ultrasound. The first study group included women aged 20–30 (n = 52) and the second group included women aged 31–40 (n = 53), with normal body weight (BMI 19–25 kg/m^2^) and normal blood pressure. Women with thyroid gland disorders were excluded based on the assessment of TSH, FT3, FT4, and ATPO levels, as well as those who had ever had a breast biopsy or had corrective breast surgery. Additional conditions for inclusion were no previous pregnancy for group 1 and giving birth to at least one child for group 2. Anthropometric measurements of body height and weight were taken for the study participants. Moreover, body composition, namely PBF (percentage body fat) and BMI (body mass index), were assessed using the method of bioelectrical impedance analysis with the use of an InBody 170 analyzer.

Subsequently, all women were subjected to double thermal imaging scans in projection: frontal plane of the front upper body, each time in a standing position with upper extremities in a 90° abduction. The camera was positioned in a straight line to the subject 1.5 m from the body. The analysis used the mean temperature value from a region of interest (ROI): the right (R) and left (L) breast, each time specifying the area of the entire breast (B) and a separate area of the nipple (N), the chest, and the abdomen. Body surface temperature measurements were performed in each case in the morning (before 11 a.m.), and all women were in the luteal phase of their menstrual cycle. The average temperature in a given body area averaged from two measurements, marked as T_mean_, was used to analyze the examination results. The measuring procedures were carried out following the standards of the European Thermographic Association [17]. According to the standards of thermal imaging tests, before taking the thermal image, the subjects rested for 15 min with their upper body uncovered in the research room to stabilize heat exchange between the body and environment. Each time the camera was turned on at least 10 min before the measurement and immediately before each subsequent measurement, it was calibrated in order to obtain the highest possible sharpness of the images.

During the imaging process, the ambient temperature and humidity were constant at the measurement site, i.e., 26 °C and 55–60%, respectively. Skin emissivity was adopted as 0.98.

A FLIR T1030sc HD camera with a detector resolution of 1024 × 678 and thermal sensitivity < 0.02 °C was used for the examinations. The FLIR Researcher ResearchIR 4, USA software was used to analyze the thermograms.

### Statistical Analysis

Statistical analyses were performed with the use of STATISTICA 11 software (StatSoft., Poland). The values of the analyzed parameters were normally distributed (which was verified with the Shapiro–Wilk test). We presented the results of the measurements as arithmetic means, standard deviations, and minimum and maximum values. To estimate the significance of differences in the temperature of selected body surface areas (breast, nipple, chest, and abdomen) and between contralateral areas within the group of women, the Student’s *t*-test was used. A comparative analysis of the significance of difference in the temperature values of selected areas between groups was carried out using the Student’s *t*-test.

Independently, in both groups, the Pearson’s test was performed to estimate the relationship between T_mean_ values of the breast, nipple, chest, abdomen temperature and age, body mass, BMI, and PBF. Additionally, the correlations between all of the examined surfaces were calculated in studied groups.

## 3. Results

The general characteristics and mean values of the anthropometric parameters of the women are presented in Table 1. The mean age of the examined women in the first group was 22.3 ± 2.04 years and was 36.4 ± 4.27 years in the second group. Despite the assumption that all of the studied women were within the range of normal body weight and BMI, the differences between the groups were significant in terms of these features, as well as the percentage of adipose tissue (Table 1), which resulted from the different ages of the subjects.

**Figure 1:**
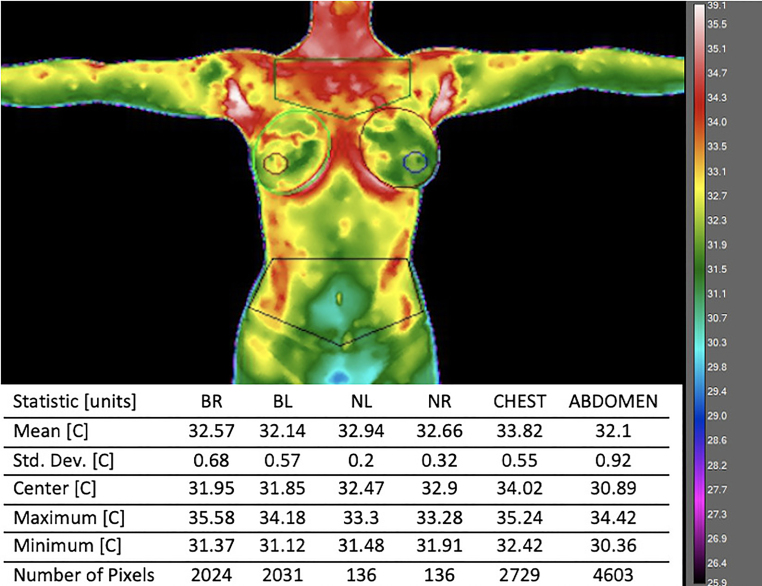
The exemplary thermal image with analyzed areas marked.

The exemplary thermal image with analyzed areas marked is presented in Figure 1.

The temperatures of the selected body surface areas are presented in Table 2. A comparative thermal analysis of the specific areas allowed us to state that the lowest temperature, regardless of age, was recorded in the abdomen areas (29.3–35.1 °C and 30.5–35.1 °C for group 1 and group 2, respectively). The highest temperatures were recorded within various areas in the studied groups; in younger women, the warmest was the nipple area (31.9–36.3 °C), and in the older women it was the chest area (32.6–35.2 °C).

In analyzing the intergroup differences in the temperature of the selected body areas (T_mean_), it was shown that, in all cases, they were statistically significantly higher in younger women.

Legend:

BR & BL—mean Tmean of both breasts
NR & NL—mean Tmean of both nipples
BR–NR—Tmean difference between the right breast and the right nipple
BL–NL—Tmean difference between the left breast and the left nipple
NR–NL—Tmean difference between the right nipple and the left nipple

A detailed thermal assessment of the breasts was carried out independently in relation to the remaining body areas of the anterior surface of the trunk and in the context of the assessment of the right and left breast thermal symmetry for both groups. It was shown that the mean values of the temperature of the entire mammary gland did not differ significantly from the temperature of the nipples, regardless of the body side and age. Statistical analysis showed no significant differences between breast and nipple temperatures in relation to the sides of the body (Table 3). However, individual assessment of contralateral thermograms showed that the differences in temperature values between the right and left breasts ranged from 0 to 1 °C and from 0 to 1.4 °C, and between the right and left nipples from 0 to 1.1 °C and from 0 to 1.5 °C for groups 1 and 2, respectively. The mean value of the temperature difference between the sides for the entire breast was 0.27 °C and 0.33 °C, and for the nipples was 0.34 and 0.31 °C for groups 1 and 2, respectively. The mammary gland in younger women was characterized by a T_mean_ value comparable to the value of T_mean_ of the chest and, at the same time, by a statistically significantly higher temperature than the abdomen area. In older women, however, T_mean_ of the breast was comparable to the T_mean_ of the abdomen and, at the same time, statistically significantly lower than the T_mean_ of the chest (Table 3).

Looking for the relationship between the temperatures of the analyzed areas and the chronological age, Pearson’s correlation was calculated for BMI and PBF in all studied women. A negative correlation was found between the T_mean_ breast and T_mean_ nipple values and age, body weight, and BMI value. However, the Pearson’s correlation coefficient r was the highest in the case of the relationship with age (−0.6168, *p* = 0.000; −0.5897, *p* =0.000, for breast and nipple, respectively). Age was also the only parameter that significantly affected the T_mean_ chest value (r = −0.3275; *p* = 0.001). The abdomen temperature was negatively correlated with age and all parameters assessing the nutritional status of the subjects (Table 4). 

**Figure 2:**
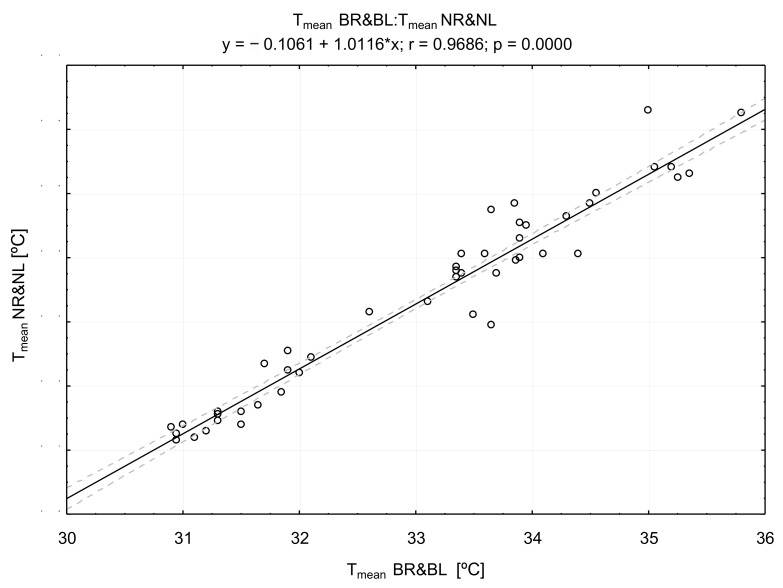
The result of the correlation between the values of breast (BR & BL) and nipple (NR & NL) temperatures (T_mean_).

**Figure 3:**
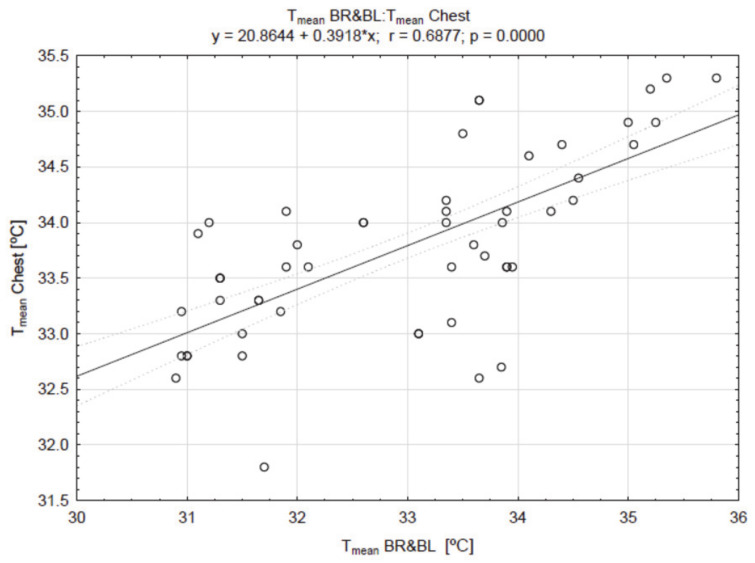
The result of the correlation between the values of breast (BR & BL) and chest temperatures (T_mean_).

**Figure 4:**
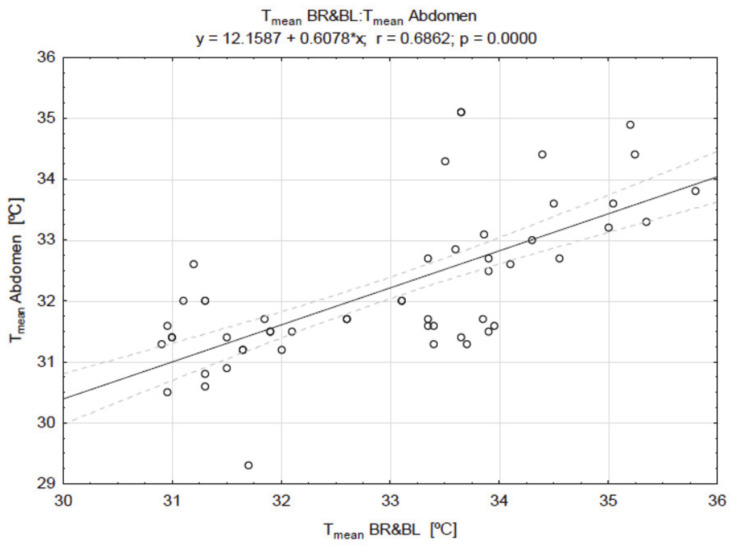
The result of the correlation between the values of breast (BR & BL) and abdomen temperatures (T_mean_).

The correlations of the breast temperature with other areas are presented in Figure 2, Figure 3 and Figure 4.

There was a very high positive correlation between the values of breast and nipple temperature (r = 0.9686; *p* =0.000) (Figure 2), as well as between the value of T_mean_ of the breast, chest (Figure 3), and abdomen (Figure 4) temperatures.

## 4. Discussion

Thermal assessment using the high-resolution digital infrared thermal imaging (thermography) method in the context of a diagnostic application is based on the non-invasive identification of body surface temperatures at a specific location and their subsequent comparative analysis with adjacent and/or symmetrical areas of the body.

Despite the use of thermographic examinations in medical science for many years, no standardized ranges of temperature values have been developed for the breast areas in healthy women.

Following these needs, the aim of the undertaken research was to characterize the distribution and range of the values of breast surface temperatures, taking into account the entire area of the mammary gland and, independently, for the nipple, in healthy women, and to assess the symmetry of the breast temperature distribution. The selection of the age range of the studied women was based on the data from the Geneva Cancer Registry, confirming the increase in the incidence of breast cancer in women aged 25–39 years by 46.7% on average per year [18].

The results of our research confirm in a statistical analysis that, in healthy women aged 20–40 years with a normative body weight and BMI value, despite individual temperature differences, the temperature distribution within the mammary gland and for the nipple area does not differ statistically significantly between the right and left sides of the body.

It should also be noted that the condition confirming the lack of thermal asymmetry based on the interpretation standards established for thermal imaging examinations is the result of the individual assessment of the value of temperature differences for contralateral areas of the body, not exceeding the value up to 0.5 °C. It should also be noted that the condition confirming the lack of thermal asymmetry based on interpretation standards established for thermal imaging examinations is the result of the individual assessment of the value of temperature differences for contralateral areas of the body, not exceeding the value up to 0.5 °C. Despite the lack of significant differences in the mathematical statistical analysis, our research showed that the individual differences in the mean values of right and left breast temperatures often exceed this value up to the maximum thermal difference of 1.4 °C for the entire breast and 1.5 °C for the nipple area, which is probably conditioned by the blood supply to the mammary glands and the high variability of individual metabolism, which does not necessarily indicate pathological changes. It is reported in the literature that the thermograms that have slight asymmetric temperature distributions signify the physiological dysfunction in patients’ breasts in most cases [19,20,21], but usually in individuals with non-tumorous breasts, the surface temperature of both breasts is close to symmetric. When there is a tumor or an abnormality, such as fibrosis, inflammation, infection, or benign condition, the symmetry is lost, and the surface temperature is altered in response to more metabolically active tumors than healthy tissue, formation of new blood vessels (angiogenesis) to sustain their accelerated growth, and growth of a more robust blood flow network [22].

The specific value of the temperature difference in the assessment of the significance of thermal asymmetry of the breast was noticed by Rassiwala et al. (2014). They showed, on the basis of a study covering 1008 female patients aged 20–60 years, that among the vast majority of them the difference in temperature of the right and left breasts did not exceed 2.5 °C (for normal clinical examination), while in 41 screened women ∆T > 3 °C; in these cases, the cause of thermal asymmetry was breast cancer confirmed with further diagnosis. Interestingly, they found only eight women with a temperature difference in the range of over 2.5 °C but less than 3 °C. Three of these were lactating mothers, and five had fibrocystic disease (fibroadenosis–4, fibroadenoma–1) [23].

Thus, apart from the assessment of thermal symmetry of contralateral breasts, changes resulting from increased metabolic activity, such as hot patches and increased nipple temperatures, may have a much greater diagnostic significance, which is also confirmed by other literature reports [24,25]. This demonstrated that abnormal thermograms included asymmetric focal hot spots, areolar and peri-areolar heat, diffuse global heat, vessel discrepancy, or thermographic edge sign, and were associated with an increased risk of breast cancer and a poorer prognosis for the breast cancer patient. Moreover, these authors suggested that, according to the comparison to the components of the Malignant Tumors (TNM) classification system, that clinical size was significantly larger (*p* = 0.006) in patients with abnormal thermograms, but such factors as age, menopausal status, and the location of tumor (left or right breast) were not related to thermographic results [25]. Based on the literature data and the results of our research, it can be assumed that interpretation of breast thermograms in a diagnostic approach should be based on the assumption that the thermal patterns in the breasts on both sides of the body under physiological conditions should be comparable, and any asymmetry of these patterns in thermograms requires further diagnosis. In our study, we compared the temperature distribution of both breasts in each of the examined women. The analysis of thermal patterns showed that the temperature of the nipple area is the highest temperature recorded in the breast area, on average by 0.39 °C for the right side and 0.45 °C for the left side in women from the younger age group, and by 0.3 °C and 0.46 °C, respectively, in the group of older women. Moreover, the temperatures of the nipple areas and the rest of the breast area correlate very high and positively under physiological conditions. Perhaps most importantly, despite the significant effect of age on the reduction of temperatures recorded from the surface of the mammary gland (mean values of 33.93 °C vs. 32.03 °C in relation to age groups) and the surface of the nipples (mean values of 34.22 °C vs. 32.30 °C in relation to age groups), the pattern of the temperature gradient between the nipple area and the entire breast remains unchanged in the age range of 20–40 years. The available literature lacks reports in which such relationships would be taken into account, and they may constitute valuable information for the use of thermal imaging in the diagnostic interpretation of neoplastic changes in the breast, including the nipple. Among the thermal features of breasts indicative of neoplastic changes, the following have been mentioned so far: highly asymmetric thermal profile, hyper-thermic vascularity, localized regions of high temperature, complex vascular patterns, areolar and peri-areolar heat patterns, and temperature differences in a single breast of more than 2 °C [22,24,25]. The results of our research allow to add to the list of these features the assessment of the temperature difference between the nipple and the rest of the breast, which, under normal conditions, should not exceed 2 °C.

It should be assumed that breast tissue, under physiological conditions, has a predictable emanation of heat patterns on the skin surface. When pathological processes occur, such as vascular disorders or inflammation, disturbances in the normal pattern occur that can be captured using a sensitive IR camera. In normal breast thermograms, symmetric heat patterns are observed in both breasts, but in the case of unilateral abnormality, asymmetry could be observed. The non-invasive, pain-free, radiation-free nature of this method, and the relatively easy access to it and its low cost compared to mammography, may direct attention to its use for breast screening, but only with the correct interpretative assumptions for symmetrical thermograms.

As mentioned earlier, among the analyzed variables, age was the most significant factor modifying the temperature of the selected breast areas, as well as body weight and BMI, although to a lesser extent. Considering the obtained data and treating them as a starting point for conclusions about the direction of further research, it seems justified to continue it with the extension of the age range of the subjects in order to verify/confirm the occurrence of physiological thermal patterns during ontogenesis, as well as their potential disturbances in women genetically at risk of breast cancer as a predictive factor. One should also take into account the differentiation of the examined women in terms of body weight and BMI.

## 5. Conclusions

Mean values of temperatures in the areas of the chest, abdomen and, above all, the breasts decrease with the age of the examined woman. Within the mammary gland, regardless of age (in the range of 20–40 years), the nipple area is characterized by the highest temperature.

The use of the high-resolution digital infrared thermal imaging method in early and screening preventive diagnosis of changes in the mammary gland requires individual interpretation of the results, taking into account the assessment of the physiological pattern of temperature distribution in both breasts. Deviations from this pattern constitute the basis for further detailed diagnostics.

## Figures and Tables

**Table 1:** Anthropometric characteristics of the studied groups.

Groups of Women	Group 1				Group 2				Student’s *t*-Test	
Parameters	M	Sd	min	max	M	Sd	min	max		
Age (yrs)	22.4	2.04	20	31	36.4	4.28	30	45		
Body height (cm)	166.8	3.99	158	174	168.3	5.49	158	180		
Body weight (kg)	63.8	4.81	55	73	67.8	4.64	60	77	−4.36	0.000 ***
BMI (kg/m^2^)	22.9	1.71	19.96	25	23.9	0.93	21.9	25	−3.58	0.001 ***
PBF (%)	26.4	3.66	16.1	32.6	28.1	3.54	21	34	−2.46	0.016 *

Statistically significant difference at * *p* < 0.05, *** *p* < 0.001.

**Table 2:** Descriptive statistics of the temperature values (T_mean_) of the selected body surface areas and the results of the significance tests of differences between the studied groups of women.

Body Surface Areas	Group 1				Group 2				Student’s *t*-Test	
	Temperature Values of Selected Body Areas (°C)									
	M	Sd	Min	Max	M	Sd	Min	Max	*t*	*p*
BR	33.97	0.901	31.7	35.8	31.95	1.189	30.9	35.4	9.76	0.0000 ***
NR	34.24	1.038	32.0	36.5	32.16	1.232	31.0	35.4	9.32	0.0000 ***
BL	33.89	1.009	31.6	35.8	32.11	1.086	30.8	35	8.67	0.0000 ***
NL	34.20	1.03	31.6	36.1	32.43	1.197	31.1	35.4	8.12	0.0000 ***
BR & BL	33.93	0.94	31.7	35.8	32.03	1.119	30.9	35.2	9.38	0.0000 ***
NR & NL	34.23	1.011	31.9	36.3	32.3	1.192	31.1	35.4	8.91	0.0000 ***
Chest	34.06	0.786	31.8	35.3	33.51	0.719	32.6	35.2	3.74	0.0003 ***
Abdomen	32.58	1.22	29.3	35.1	31.82	1.156	30.5	35.1	3.26	0.0015 **
Differences in mean temperature values between selected areas of the mammary gland (°C)										
BR–NR	0.39	0.378	0	2	0.30	0.31	0	1.2	1.48	0.14
BL–NL	0.45	0.295	0	1.1	0.46	0.31	0	1.2	–0.22	0.823
BR–BL	0.27	0.236	0	1	0.34	0.29	0	1.4	–1.30	0.196
NR–NL	0.37	0.244	0	1.1	0.31	0.41	0	1.5	0.74	0.457

Statistically significant difference at ** *p* < 0.01, *** *p* < 0.001.

**Table 3:** The results of the significance test of differences (Student’s *t*-test) between the analyzed T_mean_ values of selected areas of the female body in age groups.

Body Surface Areas	Group 1		Group 2	
Tmean (°C)	t	p	t	p
BR & BL vs. NR & NL	−1.524	0.130	−1.168	0.245
BR & BL vs. Chest	−0.755	0.452	−8.060	0.000 ***
BR & BL vs. Abdomen	6.320	0.000 ***	0.956	0.341
BR vs. BL	0.426	0.671	−0.716	0.476
NR vs. NL	0.209	0.835	−1.103	0.273
Chest vs. Abdomen	7.344	0.000 ***	9.008	0.000 ***

Statistically significant difference at *** *p* < 0.001.

**Table 4:** The results of the correlation of chronological age, body weight, BMI index, and PBF vs. T_mean_ (°C) of selected body areas.

Parameters	BR & BL	NR & NL	Chest	Abdomen
Age (yrs)	−0.6168 *p* = 0.000 ***	−0.5897 *p* = 0.000 ***	−0.3275*p* = 0.001 ***	−0.2684 *p* = 0.006 **
Body weight (kg)	−0.2154 *p* = 0.027 *	−0.2246 *p* = 0.021*	−0.016 *p* = 0.871	−0.2067 *p* = 0.034 *
BMI (kg/m^2^)	−0.224 *p* = 0.022 *	−0.2281 *p* = 0.019*	−0.0914 *p* = 0.354	−0.2634 *p* = 0.007 **
PBF (%)	−0.1417 *p* = 0.149	−0.1137 *p* = 0.248	−0.0893 *p* = 0.365	−0.288 *p* = 0.003 **

**S**tatistically significant difference at * *p* < 0.05, ** *p* < 0.01, *** *p* < 0.001.
